# Intracranial epidural hematoma after spinal anesthesia for cesarean section: a case report

**DOI:** 10.1186/s40981-024-00744-x

**Published:** 2024-10-07

**Authors:** Hiroshi Nagasaka, Yuta Horikoshi, Tina Nakamura, Hiroshi Hoshijima, Noritaka Imamachi, Katsushi Doi, Tsutomu Mieda

**Affiliations:** 1 Department of Anesthesiology, Saitama Medical University Hospital, 38 Morohongo, Moroyamacho, Irumagun, Saitama, 350-0495 Japan; 2 Division of Dento-Oral Anesthesiology, Tohoku University Graduate School of Dentistry, 4-1 Seiryomachi, Aoba, Sendai, Miyagi Japan

**Keywords:** Intracranial epidural hematoma, Cesarean section, Spinal anesthesia

## Abstract

**Background:**

Although subdural hematoma is a rare complication after spinal anesthesia, there have been no reports of an intracranial epidural hematoma after cesarean section with spinal anesthesia.

**Case presentation:**

A 32-year-old nulliparous woman at the 35^th^ week of a twin pregnancy underwent an emergency cesarean section due to her first contraction. She had no preoperative complications and the spinal anesthesia was uneventful, with 0.5% bupivacaine 12 mg and fentanyl 15 µg from the L3/4 intervertebral space. She complained of headache and nausea 15 min after spinal anesthesia, demonstrating a consciousness disturbance after surgery. Computed tomography 2 h after the cesarean section revealed an intracranial epidural hematoma. She underwent decompressive craniotomy 1 h later.

**Conclusion:**

This case highlights the possible development of an intracranial epidural hematoma in low-risk obstetric patients.

## Background

Intracranial hemorrhage is a rare but serious complication after neuraxial anesthesia that develops predominantly after dural puncture for obstetric indications in relatively young women [1]. Most cases of intracranial hemorrhage after dural puncture are subdural hematomas, and intracranial epidural hematomas are extremely rare [1–4]. To the best of our knowledge, there are no reports of an intracranial epidural hematoma after an emergency cesarean section with spinal anesthesia. We report a case of an intracranial epidural hematoma after spinal anesthesia for a cesarean section.

## Case presentation

A 32-year-old nulliparous woman at the 35^th^ week of a twin pregnancy underwent an emergency cesarean section because of her first contractions, although she was scheduled for an elective cesarean section at 37 weeks gestation. At 31 weeks gestation, she was admitted to the hospital for impending preterm labor. After admission, ritodrine was started for irregular uterine contractions. She had no particular history of trauma or habitual headache, did not suffer from hypertension or eclampsia, and was not taking aspirin or anticoagulants. After entering the operating room while crying, her vital signs and respiratory rate were normal. She was able to answer simple questions about her medical history and whether she had been eating or drinking. The spinal tap was successful at the first attempt with a 27-gauge pencil point needle from the L3/4 interspace. The cerebrospinal fluid was clear, 2.5 ml of 0.5% hyperbaric bupivacaine with 15 µg of fentanyl was injected, and a cold sensation was absent at T3. The Apgar scores were 8 and 9 points at 1 and 5 min after delivery, respectively, in both neonates. Fifteen minutes after spinal anesthesia and prior to the start of the cesarean section, she complained of nausea and a “zinging” pain from the right parietal region to the temporal region of her head. Moreover, she stated that it was the most painful headache she had ever experienced, although it was bearable. Her vital signs continued to be stable. A total of 6.6 mg of dexamethasone, 4 mg of ondansetron, 1000 mg of acetaminophen, 85 μg of fentanyl, 10 mg of metoclopramide, and 2.5 mg of droperidol were administered during the cesarean section for the treatment of intraoperative nausea and headache. After surgery, a dermatomal level of sensory block at the level of Th3 was confirmed. When the patient left the operating room, her blood pressure was 128/72 mmHg, and her heart rate was 75 bpm. No abnormalities in pupil size, left‒right differences, or position were observed. She was still able to respond to verbal instructions. She did not complain of headache or nausea and maintained a good grip. Thus, no paralysis of her upper extremities was identified that would raise suspicion of an occupying lesion of the brain.

**Fig. 1:**
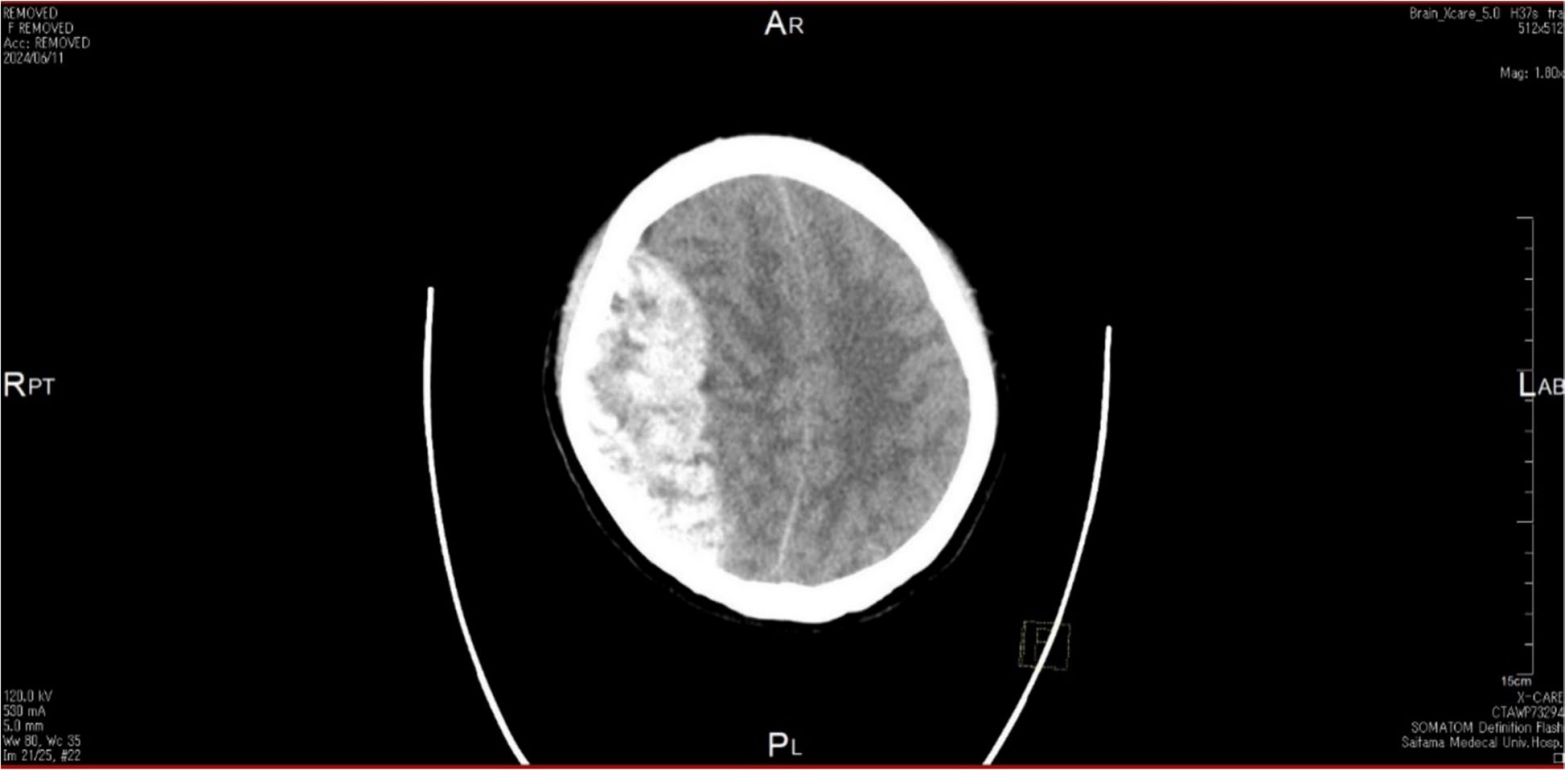
Computed tomography of the head (horizontal) after cesarean section before neurosurgery revealing an epidural hematoma with a midline shift in the right parietal region. Fracture lines are not recognized

Postoperatively, oxygen was administered via a mask at 5 l/min, but the patient exhibited somnolence. We cannot rule out the possibility that residual effects of the fentanyl and droperidol used intraoperatively were the cause of the decreased level of consciousness. A computed tomography (CT) scan 2 h after surgery revealed a right intracranial epidural hematoma (Fig. 1). At this time, her heart rate rapidly increased to 150 bpm, but her oxygen saturation was maintained at 100% due to mandibular lifting. The bilateral pupils were mydriatic, and the light reflex disappeared. The eyeballs were positioned at the midline. The extremities were flaccid, and there was slight escape behavior with pain stimulation. Tracheal intubation was performed due to respiratory arrest. Her systolic blood pressure did not exceed 140 mmHg or more during the antenatal period. Upon the diagnosis of intracranial acute epidural hematoma, an emergency decompressive craniotomy was performed 1 h later. The Glasgow Coma Scale score was 5 (eye-opening (E) 1; verbal performance (V) 1; motor response (M) 3) before surgery. The surgical findings revealed an intracranial epidural hematoma due to bleeding via the superior sagittal sinus, with no subarachnoid or subdural hematoma. The patient was extubated 4 days postoperatively. CT angiography and cerebral angiography were performed postoperatively but did not detect any abnormal vascular findings, such as aneurysms, arteriovenous fistulas, arteriovenous malformations, or sinus thrombosis. Magnetic resonance imaging also revealed no abnormal findings in the brain parenchyma, dura mater, or adjacent nasal sinuses. The patient underwent cranioplasty on the 23rd postoperative day. She had a 30-day postoperative Glasgow Coma score of 8–12 for E2–4V1M5–6, which varied from day to day, and she is currently in rehabilitation.

## Discussion

With respect to neurological complications after neuraxial anesthesia, postdural puncture headache is the most common complication, occurring in approximately 0.8% of obstetric patients [5]. Case reports indicate that major, severe, life-threatening neurologic complications, including subdural hematoma, subarachnoid hematoma, cerebral venous thrombosis, or bacterial meningitis, may occur following neuraxial anesthesia in obstetric patients [5–8]. To the best of our knowledge, there are no reports of an intracranial epidural hematoma after cesarean section with spinal anesthesia.

Most cases of intracranial acute epidural hematomas are caused by head trauma. Other conditions, such as blood system disorders and cancer metastasis, are minor causes of epidural hematomas [9]; however, none of these factors were applicable in the present case. There have been reports of idiopathic intracranial epidural hematomas with unknown causes in nonobstetric patients [10–13]. Although crying before entering the operating room might have triggered the development of an epidural hematoma before spinal anesthesia [13], such emotional hyperreactivity would not be the direct cause of the hematoma, because the patient stopped crying and was alert by the time the surgery began. Although a rapid decrease in cerebrospinal fluid pressure following opening of the cervical dura during spinal surgery may detach the dura from the skull and lead to an epidural hematoma [14], such a large volume of cerebrospinal fluid leakage would not have occurred after spinal anesthesia via a thin spinal needle. The cause of epidural hematoma could not be determined after various tests.

The prognosis is almost favorable if surgery is performed early after onset [13]. In the present case, the onset of headache was 15 min after the spinal tap, and the diagnosis of an epidural hematoma was made 3.5 h after uneventful spinal anesthesia, both of which were earlier than those reported previously [3, 4]. The interval from the onset of headache to surgery was only 4 h; however, neurological deficits remained.

In conclusion, this case, although rare, underscores the importance of the potential occurrence of intracranial epidural hematomas even in low-risk obstetric patient populations.

## Data Availability

Data sharing is not applicable to this article, as no datasets were generated or analyzed during the current study.

## Abbreviations

CT: Computed tomography
L: Lumbar
T: Thoracic
E: Eye opening
V: Verbal performance
M: Motor responsiveness
